# The current view of potentially inappropriate medications (PIMs) among older adults in Saudi Arabia: a systematic review

**DOI:** 10.3389/fphar.2023.1325871

**Published:** 2023-12-22

**Authors:** Fawaz M. Alotaibi

**Affiliations:** Pharmacy Practice Department, College of Clinical Pharmacy, Imam Abdulrahman Bin Faisal University, Dammam, Saudi Arabia

**Keywords:** potentially inappropriate medications, adverse drug reactions, older adults, geriatrics, pharmacists

## Abstract

**Introduction:** Potentially inappropriate medications PIMs are common among elderly population and becoming a global health issue. It has been associated with negative health consequences like preventable adverse drug reactions, hospitalization and mortality.

**Objectives:** To investigate the most commonly potentially inappropriate medications in older adults in Saudi Arabia. Additionally, we aim to gain insights into the typical healthcare settings where healthcare providers offer services related to PIMs.

**Methods:** This is a systematic review design using Preferred Reporting Items Systematic Reviews and Meta-Analysis (PRISMA) statement. PubMed and Google Scholar were used to search for the relevant studies using the following keywords (older adults, elderly, potentially inappropriate medications, inappropriate medications, PMIs, Saudi Arabia, Kingdom of Saudi Arabia) with no restrictions to the date of publications nor the study language.

**Results:** Only 8 studies have met our inclusion and exclusion criteria, which was most of them were cross-sectional study design (n = 6.75%) and all of them have been conducted in hospital-based settings. In addition, the prevalence of PIMs ranged from 19% to 80% depends on the site and administration of the study. We have found that proton pump inhibitors, non-steroidal anti-inflammatory drugs, aspirin, diuretics, gastrointestinal medications, and antidepressants were the most common reported PIMs in the included studies.

**Conclusion:** The prevalence of PIMs among the elderly in Saudi Arabia is notably high ranged from 19% to 80%, underscoring the need for additional research to assess the existing practices within this vulnerable demographic across various healthcare settings.

## Introduction

**FIGURE 1:**
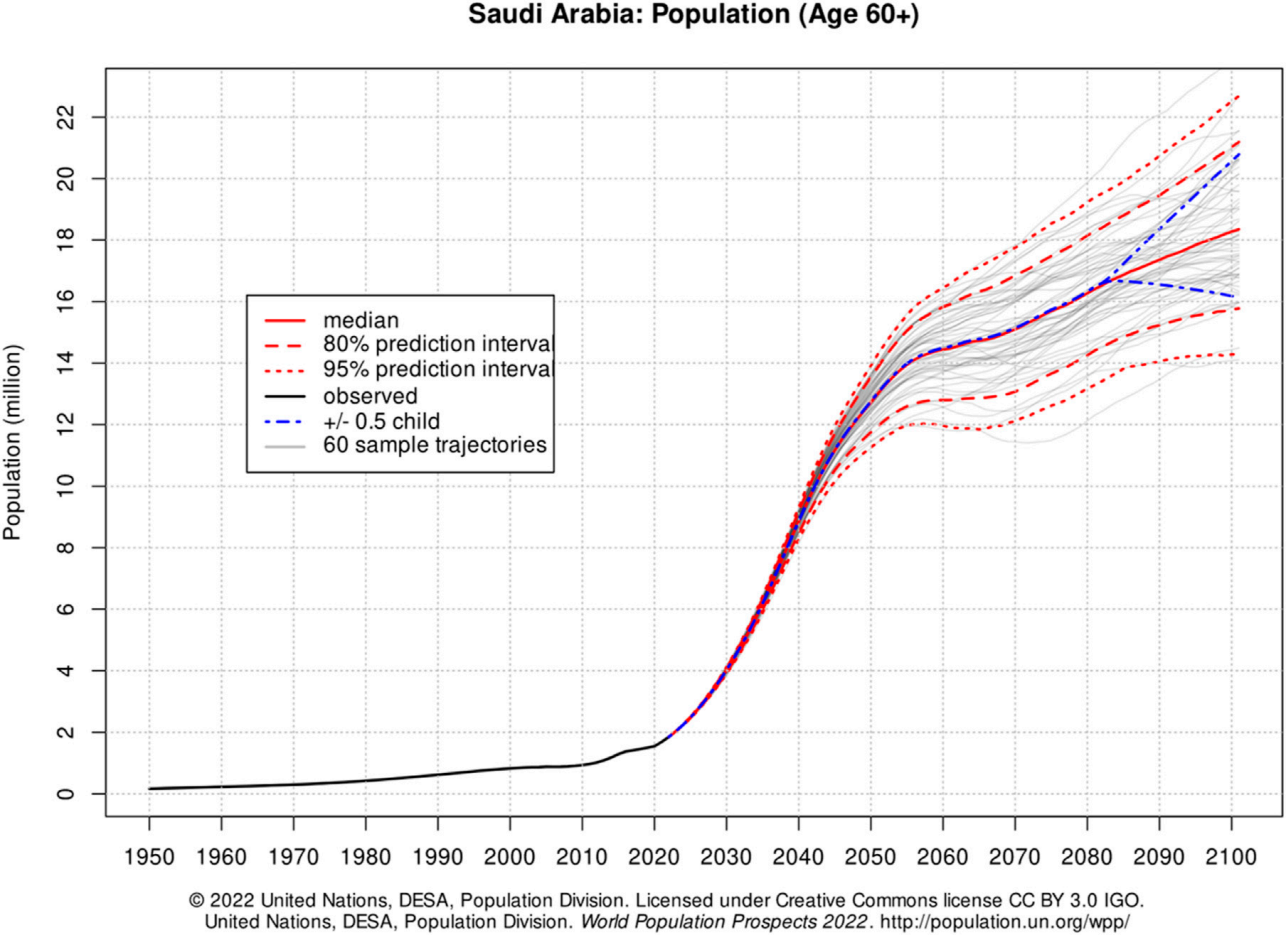
older adults’ statistics and its projection in the Kingdom of Saudi Arabia, adopted from United Nation database (United Nations Department of Economic and Social Affairs, 2020). Adapted from https://population.un.org/wpp/Graphs/Probabilistic/POP/60plus/682, access on 10.01.2023 © (copyright year) United Nations. Used with the permission of the United Nations.

Currently, there are over a million and a half elderly people (over 60 years old) living in the Kingdom of Saudi Arabia. (Basheikh et al., 2021). This number represents a 4% of the total Saudi total population. According to the recent statistics from the United Nations, this number is expected to increase to 14 million older adults, which then will represent a 16% of the total population in 2050 (Figure 1). (United Nations) This drastic change in the population age will affect the healthcare system if it is ignored or overlooked. As individuals age, their immune system undergoes a natural decline, making them more susceptible to developing multiple health conditions. Consequently, older adults often require a higher number of prescription medications (Gurwitz et al., 2003; Mangoni and Jackson, 2004; Jaul and Barron, 2017). On a global scale, the inappropriate use of medications in this demographic, known as potentially inappropriate medication (PIM) use, poses a significant risk (Akkawi et al., 2023; AlHarkan K et al., 2023). Several studies suggested that PIM use elevates the likelihood of experiencing adverse drug reactions, hospital admissions, emergency room visits, and, in severe cases, mortality. (Akkawi et al., 2023; AlHarkan K et al., 2023).In a local context, health system in Saudi Arabia is eager to change and improve the citizen health by increasing the life expectancy age to 78 by 2030 according to Saudi Vision 2030 by several programs and initiatives run by Saudi health transformational plan. Such implementation of PIM will improve health services, promoting prevention of health risk, improving quality and efficiency of the current health services towards older adults and eventually will lead to improve the health of Saudi Older adults (Chowdhury et al., 2021).

Addressing (PIM) use is a vital aspect of enhancing the wellbeing of older adults, aiming to improve their quality of life by reducing the unnecessary or harmful medications they receive. The term of PIM defined as any medication that potential risk outweigh the potential benefit when alternative is available (Matanović and Vlahovic-Palcevski, 2012). Identifying PIM depends on the tools that clinicians are using like Beer’s criteria, STOPP and START criteria which are the most commonly used in practice. Thus, PIM has been linked to negative health outcom61es such as drug-drug interactions, interactions with existing health conditions, and a higher incidence of adverse drug reactions, particularly within the older adult population (Akkawi et al., 2023). Numerous prior research investigations have documented the global prevalence of PIM use in older adult populations (Saqlain et al., 2020a). For instance, one study found that 60% of dementia patients documented having at least 1 PIMs in European countries, compared to 40% in China among similar patient population (Zhao et al., 2023). In Saudi Arabia, the prevalence of older adults who have been prescribed at least one PIM in their lifetime varies within the range of 45%–66% (Alwhaibi, 2022). The variation in this percentage is attributed to factors such as the study’s design, the specific medical conditions under investigation, or the methodologies employed to evaluate PIMs. As a result, there are various assessment tools available for evaluating PIMs, including explicit, implicit, or combined methodologies. The explicit methodology has gained widespread adoption among researchers and clinicians due to its straightforwardness and widespread recognition (Zhao et al., 2023). The widely employed explicit tools in clinical practice and prior research include the Beer’s criteria established by the American Geriatrics Society (By the 2023 American Geriatrics Society Beers Criteria^®^ Update Expert Panel, 2023), as well as STOPP (Screening Tool of Older Persons’ Prescriptions) and START (Screening Tool to Alert to Right Treatment). These tools have been the subject of examination across various settings, encompassing inpatient care, outpatient care, and the community dwelling older adults.

However, to the best of our knowledge, there is a lack of comprehensive literature on the prevalent PIMs in Saudi Arabia. Therefore firstly, this systematic review aimed to investigate the prevelance of PIMs among Saudi Older adults. Secondly, we aimed to investigate the most common healthcare settings that PIMs were happened. This effort will contribute to the existing body of knowledge and assist healthcare practitioners in Saudi Arabia in delivering the highest standard of care to the elderly population.

## Methods

### Study design

This is a systematic review design using Preferred Reporting Items Systematic Reviews and Meta-Analysis (PRISMA) statement (Page et al., 2021). The protocol is not register at PROSPERO the time of the manuscript submission. Due to the nature of our research questions, we were not able to apply the full PICO concept. However, we focused on older adult population as of the population of interest, no intervention or comparison have been looked at in our prevalence study. Our outcome of interest was the presence and the number of PIMs and in which health sittings.

### Search strategy

This systematic review was initiated in the month of August of 2023. After that, we have searched in two databases, namely, PubMed and Google Scholar. A free-text search strategy was employed, utilizing the following keywords: (older adults, elderly, potentially inappropriate medications, inappropriate medications, PMIs, Saudi Arabia, Kingdom of Saudi Arabia). In addition, Medical Subject Headings (MeSH) terms were used in PubMed like (Aged, potentially inappropriate medications, Saudi Arabia). Furthermore, in order to widen the scope of the review and make sure that we get most of the paper to answer our research question, the citation of each article has been screened for relevant articles. No restrictions were applied in terms of years of the publications or the languages.

### Inclusion and exclusion criteria

We have applied the following inclusion criteria.

1. All types of study designs were included.
2. All studies conducted in the Saudi population were included.
3. Only included those studies who focused on 60 years-old or older participants.
4. Included PIMs as one of the study main outcomes.
5. All type of diseases were covered.

At the same time, we have applied exclusion criteria as follow.

1. If the study not included list of medication names.
2. If the author has no access to the publication.

### Data extractions

The whole protocol was prepared by the main investigator and reviewed by two independent volunteer reviewers. Firstly, data were extracted by the main investigator (Alotaibi FM) and applied the first screen check on the title and abstract for eligibility by two independent volunteer reviewers. Any disagreement between the two reviewers was resolved by a third reviewer 1 (main investigator Alotaibi FM) After that, the main investigator read thoroughly all the included studies to make sure it meets the inclusion and exclusion criteria. The main author made sure that all articles had the outcomes of this study, which was PIMs prevalence, and list of PIMs. Then the data were summarized in Table 1 according to publication year, study type, study site, sample size, region in the country, study population, the most common PIMs and the tools of assessing PIM.

**TABLE 1:** Summary of the main characteristics of the included studies.

Author	Publication year	Study type	Study site	Study population	Sample size	Region in KSA
Hussain Alomar.et all Al-Omar et al., (2013)	2013	Cross-sectional study	Pharmacy Claims data (Outpatient pharmacy)	65 years of older	20,521 patients	Riyadh City
Nisreen Jastaniah.et all (Jastaniah et al., (2018)	2018	Retrospective Cohort study	Hospital Based data	60 years of older	135 patients	Jeddah City
Tariq Alhawassi. Et all Alhawassi et al., (2019)	2019	Cross-sectional study	Ambulatory care clinic in a tertiary hospital	65 years of older	4,073 patients	Riyadh City
Atheer Alturki.et all (Alturki et al., (2020)	2020	Cross-sectional study	Family Medicine Clinic	65 years of older	270 patients	Riyadh City
S.A. Alharbi et all Alharbi et al., (2022)	2022	Retrospective Cohort study	Buraidah Central Hospital	60 years of older	1,123 patients	Buraidah City
Saad Alsaad.et all (Alsaad et al., (2022)	2022	Cross-sectional study	Public tertiary hospital	65 years of older	358 patients	Riyadh City
Monira Alwhaibi Alwhaibi, (2022)	2022	Cross-sectional study	Ambulatory care clinic	65 years of older	1,853 patients	Riyadh City
Fouad Jabri.et all Jabri et al., (2023)	2023	Cross-sectional study	Outpatient clinic	65 years of older	23,417 patients	Riyadh City

## Results

**FIGURE 2:**
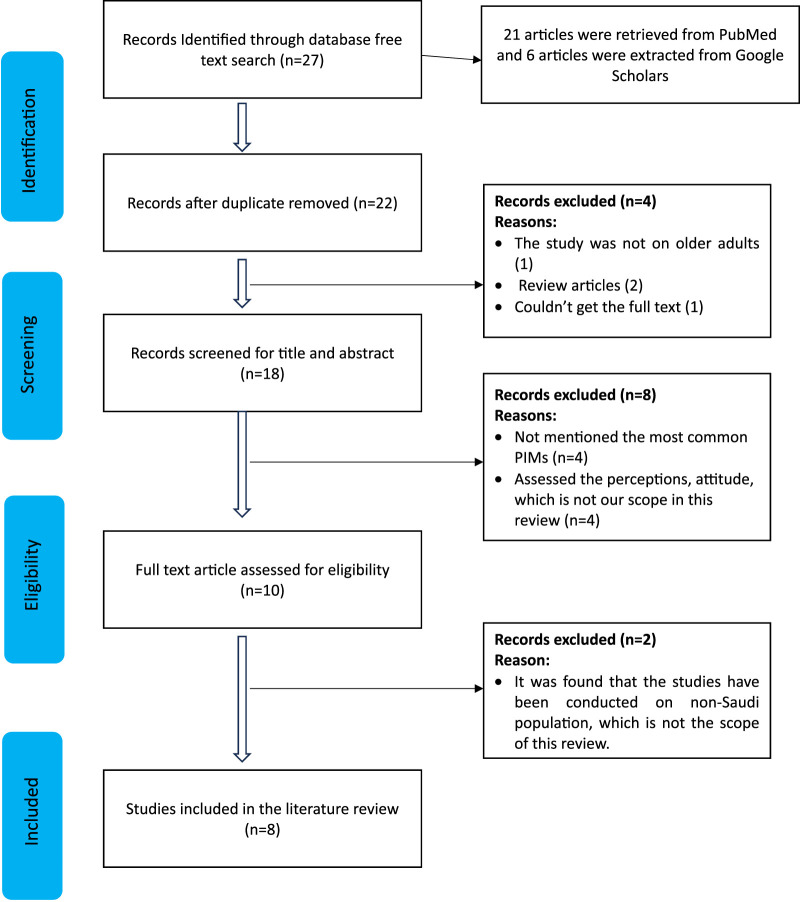
PRISMA flowchart for the identifications of the included studies.

Our initial free text search in the following databases (PubMed and Google Scholar) yielded over 3,000 articles related to PIMs. However, upon incorporating additional keywords such as “Aged,” “PIMs,” “Elderly,” “inappropriate medications,” and “Saudi Arabia,” only 27 relevant articles were identified. After removing the duplicate studies (n = 5), we ended up with 22 studies. Then we applied our inclusion and exclusion criteria, which yilded us to remove 4 studies due to due to 2 articles were review articles, 1 the author could not extract, and the last one was not on an elderly population exclusively. We ended up with 18 studies underwent an initial screening based on their titles and abstracts, during which 8 studies were excluded as they did not calculate the prevalence of PIMs or were focused on assessing the attitudes and perceptions of healthcare providers regarding PIMs, which was beyond the scope of this review. Thus, we meticulously evaluated the remaining 10 articles, and 2 of them were excluded since they did not pertain to a geriatric population in Saudi Arabia specifically the specific aim of this review. Consequently, the final number of studies included in this systematic review amounted to 8, as indicated in Figure 2. (Al-Omar et al., 2013; Jastaniah et al., 2018; Alhawassi et al., 2019; Alturki et al., 2020; Alsaad et al., 2022; Alwhaibi, 2022; Alharbi et al., 2022; Jabri et al., 2023).

The studies we examined were all published between 2013 and 2023, signifying the relatively limited attention given to this specific subject concerning the geriatric population in Saudi Arabia. This highlights the need for further research efforts to explore such phenomena in more depth. Additionally, it is noteworthy that 6 out of the included studies (constituting 75%) (Al-Omar et al., 2013; Alhawassi et al., 2019; Alturki et al., 2020; Alsaad et al., 2022; Alwhaibi, 2022; Jabri et al., 2023) focused on the elderly population, typically defined as individuals aged 65 years or older the standard definition of elderly in the US or United Kingdom studies. The two remaining studies (Alharbi et al., 2022; Jastaniah et al., 2018) considered a broader age category of 60 years or older, a specification more relevant to the region due to economic, life expectancy, retirement age, and health-related distinctions between the Saudi population and older adult populations in the United Kingdom or US. Regarding the study locations, the majority, 6 out of 8 (75%) of the included studies, were carried out in the capital city, Riyadh (Central Region). (Al-Omar et al., 2013; Alhawassi et al., 2019; Alturki et al., 2020; Alsaad et al., 2022; Alwhaibi, 2022; Jabri et al., 2023). One study was conducted in Jeddah (Western Saudi Arabia) (Jastaniah et al., 2018), and the last one took place in Buraidah City (Alharbi et al.), also situated in the Central Region. Table 1 (Al-Omar et al., 2013; Jastaniah et al., 2018; Alturki et al., 2020; Saqlain et al., 2020a; Alsaad et al., 2022; Alwhaibi, 2022; Alharbi et al., 2022; Jabri et al., 2023).

The majority of the studies included in the analysis, specifically 6 out of 8 (constituting 75%) (Al-Omar et al., 2013; Alhawassi et al., 2019; Alturki et al., 2020; Alsaad et al., 2022; Alwhaibi, 2022; Jabri et al., 2023), adopted a cross-sectional study design. The remaining 2 studies (Alharbi et al., 2022; Jastaniah et al., 2018), on the other hand, followed a retrospective cohort study design. Additionally, it is worth noting that all the studies incorporated in this review were based on data sourced from hospitals and were derived from single-center studies. Most of the examined studies had relatively short observation periods and featured small sample sizes when compared to the overall geriatric population in Saudi Arabia. Among the eight studies, three of them stood out for having larger sample sizes (Al-Omar et al., 2013; Alhawassi et al., 2019; Jabri et al., 2023), each comprising over 2,000 patients. This was primarily due to their inclusion criteria, which extended over a period of more than 1 year to provide a more comprehensive understanding of the issue. It is worth mentioning that noon of the study used multicenter approach to examine MTM services, nor any of the study used MTM as an intervention in their study.

Notably, all the studies we incorporated in this analysis employed the Beers’ criteria to evaluate PIMs in the older Saudi adult population as shown in Table 2. The Beers’ criteria is a widely recognized and validated set of guidelines established by the American Geriatrics Society back in 1991. (By the 2023 American Geriatrics Society Beers Criteria^®^ Update Expert Panel, 2023). The AGS Society routinely revisits and updates this list every 3 years, with the most recent version being released in 2023. It is noteworthy that none of the studies we reviewed utilized alternative criteria, such as STOPP, START, or other judgment-based criteria, to assess PIMs among older adults in Saudi Arabia. In addition, the most common predisposing risk factors of PIMs we have observed were comorbidities, polypharmacy, being an older person and frailty.

**TABLE 2:** PIMs tools and its predicting risk factors of having at least one PIM.

Author	Tools to detect PIMs	Predisposing risk factors of the probability of having at least one PIMs
Hussain Alomar.et all Al-Omar et al., (2013)	Beer’s criteria 2003	Family, community medicine and cardiology department
Nisreen Jastaniah.et all Jastaniah et al., (2018)	Beer’s criteria 2012	Patient admitted to the surgical wards and medical wards
Tariq Alhawassi. Et all Alhawassi et al., (2019)	Beer’s criteria 2015	Older adults with DM, HF, IHD, CKD, cnacer, osteoarthritis, osteoporosis and polypharmacy
Atheer Alturki.et all Alturki et al., (2020)	Beer’s criteria 2015	Older adults, being female, HTN, osteoarthritis and polypharmacy
S.A. Alharbi et all Alharbi et al., (2022)	Beer’s criteria 2019	Comorbidities and polypharmacy
Saad Alsaad.et all Alsaad et al., (2022)	Beer’s criteria	Polypharmacy, comorbidities and frailty score
Monira Alwhaibi Alwhaibi, (2022)	Beer’s criteria 2019	65 years of older
Fouad Jabri.et all Jabri et al., (2023)	Beer’s criteria 2019	65 years of older

As indicated in Table 3 the prevalence of PIMs identified in these studies we examined included proton pump inhibitors, non-steroidal anti-inflammatory drugs, aspirin, baclofen, diuretics, gastrointestinal medications, and antidepressants. One study examined the association between PIM and other diseases, and they found a positive association between PIMs and hypertension, diabetes. (Alhawassi et al., 2019). In our study we observed that all patients with polypharmacy (taking 5 medications or more) are at higher risk of having at least one PIM. (Al-Omar et al., 2013; Alhawassi et al., 2019; Alwhaibi, 2022). Moreover, all the included studies have calculated the prevalence of PIMs in their patient population, which ranged from 19% to 80% of the participants have at least one PIMs. All studies focused on prescription medications, while there are some of OTC medications cannot be ignored in this context. The high occurrence of these common medications can be attributed to healthcare providers frequently prescribing aspirin for the primary prevention of heart disease, despite a lack of scientific evidence supporting this practice. (Alghadeer et al., 2022). Additionally, proton pump inhibitors are commonly prescribed to safeguard the stomach when multiple medications are involved, even though PPIs themselves may elevate the risk of internal bleeding and clostridium difficile infection. (AlKhamees et al., 2018).

**TABLE 3:** Summary of the calculated prevalence of PIMs in the included studies.

	Hussain Alomar.et all Al-Omar et al., (2013)	Nisreen Jastaniah.et all Jastaniah et al., (2018)	Tariq Alhawassi. Et all Alhawassi et al., (2019)	Atheer Alturki.et all Alturki et al., (2020)	S.A. Alharbi et all Alharbi et al., (2022)	Saad Alsaad.et all Alsaad et al., (2022)	Monira Alwhaibi Alwhaibi, (2022)	Fouad Jabri.et all Jabri et al., (2023)
The calculated prevalence of PIM	43.6%	80%	57.6%	60.7%	66.25%	45.8%	56%	63.6%
The most common PIMs were found	Digoxin, ferrous, dipyridamole, amitriptyline and amiodarone	Insulin, NSAID and Vasodilators	Gastrointestinal agent, endocrine agent, diuretics and antidepressants	PPI, diuretics, NSAID and aspirin	NSAID, PPI, baclofen and diuretics and aspirin	Amitriptyline, olanzapine, NSAID and diltiazem	Gastrointestinal and endocrine agent	Aspirin, pantoprazole, levothyroxine, insulin Glargine and meloxicam

NSAID = non-steroidal anti-inflammatory drugs; PPI = proton pump inhibitors.

## Discussion

The prevalence of PIMs use among older adults is a significant global concern. This issue is largely attributed to the intricate process of aging, the widespread use of multiple medications (polypharmacy), the presence of concurrent medical conditions (comorbidities), consultations with multiple healthcare providers, and the level of health literacy in patients. (Daunt et al., 2023). This pervasive problem amplifies the risk of hospitalization, emergency department visits, and mortality among the elderly. Consequently, it is of utmost importance to gain a comprehensive understanding of the current occurrence of PIMs within the geriatric population of Saudi Arabia and to explore various approaches to mitigate this challenge.

The current systematic review reveals that the majority of the included studies involved patients aged 65 years or older, as opposed to the more reasonable age range of 60 years or older. Considering the retirement age of 60 and the average life expectancy in Saudi Arabia, which remains around 70, geriatrics studies in the kingdom should be among 60 years of older. Adopting this broader age definition could have allowed under representation of the results. Thus, this could serve as a recommendation for future studies seeking to investigate similar topics in the geriatric population of Saudi Arabia. Consequently, the studies presented in this review may have potentially underestimated the prevalence of PIMs by recruiting fewer older adults than anticipated. It is worth noting that all the studies relied on hospital-based data, and none of them were conducted prospectively, either directly within the community or within primary care physician offices. This highlights the inherent challenges of conducting research in this population. Securing funding from entities such as the Ministry of Health, Ministry of Education, and the recently established Research Development and Innovation Authority is imperative. Especially given that research related to aging is one of the officially announced priorities in research for these Saudi government institutions. Another noteworthy observation made in the course of this review is the distinct practice of geriatricians and pharmacists specializing in geriatrics. Most of the studies included in our review were investigated or authored by pharmacists, which is unsurprising given their natural affinity for the subject matter and given their expertise in medications. It is advisable that both areas of expertise collaborate to provide valuable insights in a single research endeavor. This collaborative approach is particularly essential in this evolving field that demands further exploration in the future. Which will enhance the overall health-related quality of life for the geriatric population in Saudi Arabia.

Another intriguing discovery from our systematic review is that none of the studies under consideration focused on long-term care residents. All the study participants were either inpatients or outpatients visiting government hospitals. Long-term care in Saudi Arabia differs from that in the United States or other European countries, as not all residents are older adults. Some of long term residents in KSA may include long-term bedridden patients due to road car accidents, individuals with paralysis, or older adults (Al-Shammari et al., 1997). Long-term care residents possess distinct characteristics that make them susceptible to polypharmacy, comorbidities, limited health literacy, lack of family support, and an increased likelihood of PIMs.

Regrettably, long-term care residents in Saudi Arabia are often unintentionally overlooked, partly due to cultural factors that discourage placing older adults in such facilities, with feelings of shame and guilt associated with this choice. Therefore, it becomes the responsibility of geriatric researchers from all fields to comprehensively investigate all health-related issues pertinent to this vulnerable population residing in long-term care facilities in Saudi Arabia.

Remarkably, all the studies included in our analysis exclusively employed Beer’s criteria to investigate PIMs among older adults. While Beer’s criteria are widely used in practice, it is important to recognize that alternative criteria such as STOPP, START (Díaz Planelles et al., 2023), or Medication Therapy Management (MTM) services can also be utilized (Lin et al., 2018). MTM is a service provided by pharmacists or licensed healthcare professionals to older adults, involving a comprehensive review of their medication regimens. Research has established that MTM services are effective in reducing adverse drug reactions, drug-drug interactions, and lowering mortality rates (Caffiero et al., 2017). It is advisable to consider using criteria other than Beer’s list to corroborate previous research findings and to identify the best practices that can be universally applied across geriatric clinics, ensuring the protection of older adults from preventable, harmful side effects resulting from medication misuse.

Some noteworthy findings emerged from our study, revealing a wide range in the prevalence of PIMs among Saudi geriatric individuals, spanning from 19% to 80%. This variation is in line with earlier research, and it can be attributed to factors such as the study’s location, the specific Beer’s criteria used, and variations in how age is defined. Furthermore, a multicentre study by Parekh et al. (Parekh et al., 2019) indicated that 22% of their participants had at least one PIM at the time of hospital discharge. Another investigation by Blanco-Reina et al. (Blanco-Reina et al., 2015) compared two commonly used tools, STOPP and Beer’s criteria, and found that 48% of PIMs were detected using STOPP criteria, while 54% were identified when Beer’s criteria were applied, aligning with our findings from this review.

In our systematic review, the most prevalent PIMs were proton pump inhibitors (PPI), non-steroidal anti-inflammatory drugs (NSAIDs), antidepressants, and diuretics, which corresponds with the outcomes of prior studies with similar objectives (Tian et al., 2022; Zhao et al., 2023). We recommend the implementation of educational programs for all healthcare providers directly involved in the care of older adults. Similarly, community pharmacists in Saudi Arabia should receive education on Beer’s criteria and the most harmful medications for older adults. Developing a mobile application that encompasses all criteria, including STOPP, START, or Beers, and making it accessible to the public for cautious use, could be a valuable endeavor. We believe that these recommendations will contribute to mitigating the prevalent issues faced by older adults in Saudi Arabia, ultimately supporting the realization of one of the key objectives of Saudi Vision 2030, the healthcare transformation plan for 2025 (Chowdhury et al., 2021; Vision, 2030).

Our systematic review indicated that PIMs is prevalent in our region among older adults’ population, a threat that needs further investigations and initiatives. For instance, increasing the role of clinical pharmacy who specialized in geriatrics would help to fill this gap by conducting medication therapy management in a regular basis whether on an inpatient or outpatient level. In addition, the results of our study would help policymakers to incorporate medication therapy managements MTMs services in community pharmacy settings to cover wider patient population. Such services will help reduce unncessarly medication, thus eventually will help to increase patients’ quality of life.

Our study findings will enrich the literature to support the idea of implementing medication therapy management MTM services among older adults’ population. MTM services that will run by the pharmacists will reduce the unneeded prescription medication, which eventually will reduce the adverse drug reaction, emergency department visits due to medication side effect, and medication associated mortality. Thus, it will reflect on the health-related quality of life to the patient and caregivers and will reduce the direct and indirect cost of each patient to the government or insurance company. As a results, this will help to achieve the goals of health transformational plan, a pillar in the Saudi Vision 2030.

We want to acknowledge that our study comes with certain limitations. Firstly, our search was confined to just two databases, PubMed and Google Scholar, which are extensively used in health sciences and contain a vast number of publications. Secondly, our primary emphasis was on presenting the prevalence data and the most frequently reported PIMs, without delving into the assessment of the evidence’s quality—a specific aspect that fell outside the scope of this review. Lastly, while volunteer reviewers were engaged in evaluating the initial screening (comprising titles and abstracts) for initial inclusion, there remains the potential for selection bias, stemming from the fact that the main investigator was solely responsible for this review.

## Conclusion

The prevalence of PIMs among the elderly in Saudi Arabia is notably high, underscoring the need for additional research to assess the existing practices within this vulnerable demographic across various healthcare settings. The most frequently reported PIMs in the studies we included were proton pump inhibitors, antidepressants, and aspirin, highlighting the importance for geriatric healthcare professionals to delve deeper into these issues. Future research directions should focus on investigating the prevalence of PIMs within long-term care facilities in Saudi Arabia, employing diverse assessment tools. Furthermore, it is imperative to conduct a comprehensive study encompassing all regions of Saudi Arabia to gain a comprehensive and more broadly applicable understanding of the entire older adult population residing in the country.

## Data Availability

The original contributions presented in the study are included in the article/Supplementary material, further inquiries can be directed to the corresponding author.
